# Tristetraprolin, Inflammation, and Metabolic Syndrome in Arab Adults: A Case Control Study

**DOI:** 10.3390/biology10060550

**Published:** 2021-06-18

**Authors:** Nasser M. Al-Daghri, Albatul Y.A. Al-Shuwaie, Amani Alghamdi, Osama E. Amer, Malak N.K. Khattak, Mohammed G.A. Ansari, Abdullah M. Alnaami, Shaun Sabico

**Affiliations:** Chair for Biomarkers of Chronic Diseases, Biochemistry Department, College of Science, King Saud University, Riyadh 11451, Saudi Arabia; albatoolyousef16@gmail.com (A.Y.A.A.-S.); aalghamedi@ksu.edu.sa (A.A.); osamaemam@gmail.com (O.E.A.); malaknawaz@yahoo.com (M.N.K.K.); ansari.bio1@gmail.com (M.G.A.A.); aalnaami@yahoo.com (A.M.A.); eaglescout01@yahoo.com (S.S.)

**Keywords:** tristetraprolin, Saudi, metabolic syndrome, inflammatory markers, insulin

## Abstract

**Simple Summary:**

Metabolic syndrome (MetS) is a common disorder characterized as a low-grade chronic inflammatory state. The association of tristetraprolin (TTP), a novel anti-inflammatory protein, and MetS remains to be explored. We evaluated circulating TTP in a group of adult males and females with and without MetS. Serum levels of TTP were higher in the MetS group than in controls. In all subjects, serum TTP was also correlated with MetS components (e.g., glucose, lipids, and obesity indices). These findings suggest that TTP may be a promising biomarker for MetS.

**Abstract:**

Tristetraprolin (TTP) is an mRNA binding protein suggested to have a substantial role in regulating the mRNA expression of numerous inflammatory factors, but data on TTP and its association with metabolic syndrome (MetS), a chronic low-grade inflammatory disorder, are scarce. We hypothesize that TTP may modulate MetS and its components. A total of 200 Saudi adults (aged 38.6 ± 8.3 years) were included in this cross-sectional study. Anthropometrics data were collected and fasting blood glucose taken for the assessment of glycemic, lipids and inflammatory markers using commercially available assays. The National Cholesterol Education Program Adult Treatment Panel (NCEP ATP III) criteria were used to define MetS. Results showed significantly higher levels of TTP in the MetS group than in controls [288.1 pg/mL vs. 150.9 pg/mL, *p* < 0.001]. Circulating TTP was significantly associated with tumor necrosis factor alpha [TNF-α, R = 0.30, *p* < 0.05], interleukin 1β [IL-1β, R = 0.41, *p* < 0.01] and C-reactive protein [CRP, R = 0.36, *p* < 0.01], adiponectin [R = 0.36, *p* < 0.05], insulin [R = 0.37, *p* < 0.05], and insulin resistance [HOMA-IR, R = 0.40, *p* < 0.05]. Receiver operating characteristics (ROC) suggest a potential use of TTP as diagnostic biomarker for MetS [AUC = 0.819, *p* < 0.001]. The findings suggest that TTP is associated with inflammation and glycemia, which may influence MetS. TTP is a promising diagnostic biomarker for MetS which can be confirmed in larger cohorts.

## 1. Introduction

Metabolic syndrome (MetS) is a common disorder secondary to unhealthy diet and lack of physical activity. If left untreated, it can predispose to cardiovascular diseases and type 2 diabetes (T2D) [1,2,3]. Currently, there are four commonly used operating definitions of MetS: the European Group for Study of Insulin Resistance (EGIR), International Diabetes Foundation (IDF), National Cholesterol Education Program (NCEP), Adult Treatment Panel III (ATP III), and World Health Organization (WHO) [4]. These definitions have relatively the same components and mostly involve impaired glucose tolerance (IGT), obesity, dyslipidemia, and hypertension [4]. In Saudi Arabia, obesity-mediated MetS and insulin resistance (IR) are increasingly heavy burdens in society, with widespread economic impact [5,6]. As of 2018, the prevalence of MetS in Saudi Arabia was reported to be 31.6% based on the IDF criteria and 39.8% according to NCEP ATP III criteria [7].

MetS is considered as a chronic low-grade inflammatory state evidenced by elevated levels of several inflammatory biomarkers [e.g., C-reactive protein (CRP)] and cytokines [e.g., interleukin 1 beta (IL-1β) and tumor necrosis factor alpha (TNF-α)] [8,9]. Therefore, a wide research is being dedicated to identifying the chronic inflammation stimuli in MetS. Several studies have determined that the main initiator sites of inflammation in MetS are adipose depots, intestine, and liver [10,11,12,13], considered as a metabolic stress response due to chronic caloric excess and subsequent cell death [14,15,16]. The released inflammatory factors from one site can induce inflammation in other tissues, consequently increasing the chronic inflammatory state and generalized tissue dysfunction/damage [12]. Understanding the modulation of this inflammatory state could assist in ameliorating the detrimental effects of MetS and its associated consequences. One way to achieve this is by investigating novel biomarkers which, based on their functional properties, can potentially link the underlying inflammation seen in MetS and its components and may serve as a diagnostic marker. Among the roster of promising biomarkers which remain hitherto under investigated is tristetraprolin.

Tristetraprolin (TTP), or zinc-finger protein 36 (ZFP36), is a well-characterized zinc finger-containing RNA-binding protein, acting as a post-transcriptional regulator of immune functions through binding to the adenosine and uridine (AU)-rich elements (AREs) of the mRNAs 3′ untranslated regions (3′ UTRs), and recruits deadenylase complexes leading to the degradation of its target mRNAs [17,18]. As an mRNA decay activator protein, studies have established that TTP/ZFP36 binds to AREs of several inflammatory factors, like interlukin-6 (IL-6), IL-23 [19], TNF-α [20], IL-10 [21], CXCL1, CXCL2 [22], IL-17 [23], and CCL3 [24], resulting in the degradation of mRNA. TTP not only destabilizes mRNAs, but also inhibits the process of mRNA translation [25,26,27]. Recently, Patial et al. [28] have found a protective effect of the overexpressed TTP against chronic immune-mediated inflammatory diseases (e.g., autoimmune encephalomyelitis, psoriasis, and arthritis). Since TTP is considered as an endogenous anti-inflammatory protein involved in various physiological and pathological processes, its role in the inflammatory state associated with MetS merits investigation. Hence, the present cross-sectional study investigated, for the first time, the differences in serum levels of TTP in adults with or without MetS and its relationship with inflammatory markers and MetS components.

## 2. Methods

### 2.1. Study Design and Participants

A total of 200 Saudi adult males and females aged 38.6 ± 8.3 years (100 with MetS and 100 without MetS as control group) were randomly selected from the master database of the Chair for Biomarkers of Chronic Diseases (CBCD) in King Saud University (KSU, Riyadh, KSA), which contains information and blood samples of Saudi participants aged 1–65 years who participated in the capital-wide epidemiologic studies on chronic diseases [5,6,28,29]. The inclusion criteria were consenting males and females, >35 years old, and the exclusion criteria those participants with malignancy, cardiac or lung diseases, etc., that required immediate medical attention. Ethical approval was obtained from the Ethics Committee of the College of Science (No. 8/25/454239).

### 2.2. MetS Components Classification of Participants

Participants were screened for MetS using the NCEP ATP III criteria [30], which indicates that a person has MetS when at least three of the following five risk factors are present:

(1) Waist circumference (Central obesity) of >101.6 cm in males and >88.9 cm in females.
(2) Fasting glucose (Hyperglycemia) >5.6 mmol/L.
(3) Low high density lipoprotein cholesterol (HDL-c); for males <1.03 mmol/L and for females <1.30 mmol/L.
(4) Fasting triglycerides (Hypertriglyceridemia) >1.7 mmol/L.
(5) Hypertension; diastolic blood pressure >85 mmHg and/or systolic blood pressure >130 mmHg.

### 2.3. Anthropometrics and Biochemical Analyses

Anthropometric measures were extracted from the database, and blood samples were retrieved from the biobank. Body mass index (BMI) was calculated (kg/m^2^). Fasting glucose and lipid profile were analyzed using a chemical analyzer (Konelab, Espoo, Finland). TNF-α, IL-1β, leptin, and adiponectin were measured using multiplex assay kits that utilized the Luminex xMAP Technology platform (Luminex Corporation, Austin, TX, USA). Serum CRP and TTP levels were assessed using an enzyme-linked immunosorbent assay (ELISA) according to the manufacturer’s instructions. The intra-assay %CV was TNF-α: 2.6, IL-1β: 2.3, leptin: 4.6, adiponectin: <10, CRP: 3.8, TTP: <8%. The inter-assay %CV was TNF-α: 13, IL-1β: 6.7, liptin: 8, adiponectin: <15, CRP: 7, TTP: <10. Insulin was measured using Liaison XL (DiaSorin, Saluggia, Italy). HOMA-IR was computed as follows: fasting insulin (μIU/mL) × fasting glucose (mmol/mL)/22.5. HOMA-B was calculated using the following formula: 20 × fasting insulin (μIU/mL)/fasting glucose (mmol/mL) − 3.5.

### 2.4. Statistical Analysis

Data were analyzed using SPSS (version 22, Chicago, IL, USA). Using G*power calculations for power analysis, a TTP effect size = 0.14 was observed between controls and MetS using a sample size of 200. The margin of error = 0.05, and the actual power achieved = 86.1%. Continuous data were presented as mean ± standard deviation (SD) for normal variables, and non-Gaussian variables were presented in median (1st and 3rd) percentiles. All continuous variables were checked for normality using the Kolmogorov–Smirnov test. Non-Gaussian variables were log-transformed prior to parametric analysis. An independent *t*-test and a Mann–Whitney U were performed to compare the mean and median differences in Gaussian and non-Gaussian variables. A linear regression analysis was performed to determine the explained variation and correlation between TTP and MetS components after adjusting for age and gender. Correlations between variables were done using a Pearson’s and a spearman correlation analysis. A sensitivity and specificity analysis was performed for TTP with MetS. *p* value <0.05 was considered statistically significant. All figures were plotted in MS Excel except for the receiver operating characteristics (ROC) curve. The ROC curve was plotted in MedCalc^®^ Statistical Software version 19.5.2 (MedCalc Software Ltd., Ostend, Belgium; https://www.medcalc.org; accessed on 20 May 2021). The curve was performed to optimize cutoff points for TTP based on high sensitivity and specificity, PPV and ± LR, using independent group design (condition variable MetS) and the criterion variable TTP, with a higher value indicating a positive condition. The area under the curve (AUC) was also calculated to provide a comparison of the performance of TTP as a biomarker. An AUC of 0.9 to 1 is considered excellent, 0.8 to 0.9 is considered good, 0.7 to 0.8 is considered fair, 0.6 to 0.7 is considered poor, and 0.5 to 0.6 is considered very poor.

## 3. Results

### 3.1. General Comparison between MetS Patients and Controls

A total of 200 Saudi adult participants were recruited (100 control and 100 with MetS). Table 1 shows the clinical characteristics of the study participants. The mean ages for controls and MetS were 35 and 41 years, respectively, *p* < 0.001. TTP was significantly higher in the MetS group than in the control group (*p* < 0.001). Similarly, the inflammatory markers were significantly higher in the MetS group than in the control group, TNF-α (*p* < 0.001), IL-1β (*p* < 0.001), and CRP (*p* < 0.001). For the anthropometric and other clinical characteristics, the MetS group, as expected, had a significantly higher mean waist circumference, systolic blood pressure (SBP), diastolic blood pressure (DBP), fasting blood glucose, and triglycerides (*p* values < 0.001]. High-density lipoprotein cholesterol (HDL-c) was significantly lower in the MetS group than in the control group (*p* < 0.001). The differences in anthropometrics and the general characteristics of the study participants are shown in Supplementary Table S1, showing, as expected, a significantly higher BMI, waist, waist-hip ratio, blood pressure, glucose, and triglycerides, with a significantly lower HDL-cholesterol, in the MetS group as compared to controls (all *p*-values < 0.001 adjusted for age).

### 3.2. Comparison between Male and Female Participants in Both Groups

Table 2 shows the clinical characteristics of the study participants by sex. Results showed that TTP, TNF-α, IL-1β, and CRP were significantly higher in MetS patients than controls, in both sexes. As expected, all MetS component parameters were higher in MetS patients in both sexes than in controls. MetS patients had a significantly higher waist circumference than control participants, as well as a higher BMI, SBP, DBP, glucose, total cholesterol, and triglycerides. HDL-c was significantly lower in MetS patients than in the control participants, in both sexes.

### 3.3. Relationship between TTP and Individual Components of MetS

**Figure 1:**
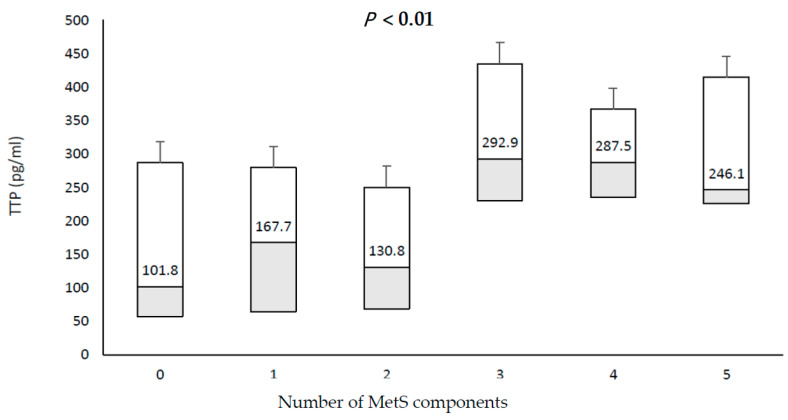
Comparison of serum TPP levels between the individual MetS component in MetS patients and healthy controls.

In all subjects, TTP levels were trending higher in subjects with increasing presence of MetS components (*p* < 0.01), Figure 1.

### 3.4. Associations of TTP with Anthropometrics and Clinical Characteristics

**Figure 2:**
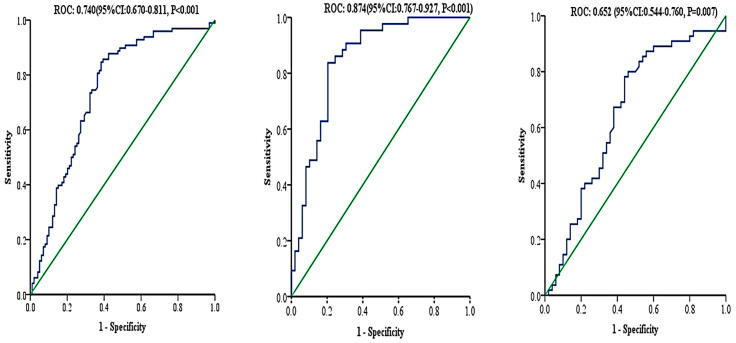
Receiver operational curve for TTP. All subjects (**left**), Male subjects (**center**), Female subjects (**right**).

TTP had a significant positive correlation with TNF-α [R = 0.18, *p* < 0.05], IL-1β [R = 0.31, *p* < 0.01], and CRP [R = 0.18, *p* < 0.05] in all participants, which persisted only in male participants after stratifying for gender, and not in females (Supplementary Table S2). For the MetS components, TTP had an inverse significant relationship with weight [R = −0.47, *p* < 0.01], BMI [R = −0.31, *p* < 0.05], and HDL-c [R = −0.48, *p* < 0.01], and a significant positive relationship with adiponectin [R = 0.36, *p* < 0.05], insulin [R = 0.37, *p* < 0.05], and HOMA-IR [R = 0.40, *p* < 0.05] only in males with MetS (Figure 2). In females with MetS, no correlation between TTP and the other study parameters was detected.

Table 3 shows the results of the multiple linear regression for the TTP as dependent variable and gender and metabolic components as independent variables. The adjusted R-squared showed that 16.4% of the variability in the concentration of TTP can be explained by the patient’s gender and metabolic components. Further, being male is negatively associated with serum TTP levels, while high blood glucose, low HDL-c, and high triglycerides are positively associated with serum TTP levels.

Lastly, an ROC analysis was performed to assess if TTP could be a viable biomarker for MetS. The area under the curve (AUC) showed that TTP could be a fair biomarker for MetS diagnosis and that its viability is higher in males than females [(All MetS patients; AUC = 0.743, Optimal cutoff ≥ 199.7, sensitivity = 87.6%, specificity = 59%, PPV = 68%, Youden index = 0.464, LR+ = 2.12, and LR = 0.210, *p* < 0.001), (female MetS patients; AUC = 0.683, Optimal cutoff ≥ 237.3, sensitivity = 79.6%, specificity = 56%, PPV = 66.2%, Youden index = 0.356, LR+ = 1.81, and LR− = 0.364, *p* < 0.001), (male MetS patients; AUC = 0.819, Optimal cutoff ≥ 207.3, sensitivity = 83.7%, specificity = 76.1%, PPV = 76.6%, Youden index = 0.60, LR+ = 3.50, and LR− = 0.214, *p* < 0.001)], Figure 2.

## 4. Discussion

The present study investigated the serum levels of TTP in a group of MetS patients compared to control individuals. Additionally, we evaluated the relationship between TTP and inflammatory markers in both groups of our cohort. Interestingly, the results of the ROC analysis indicated an important role for TTP in MetS and that TTP could be a promising diagnostic biomarker for MetS, particularly in males. Circulating TTP is altered under pathological conditions and is considered as an endogenous anti-inflammatory protein [8,9,25]. Inflammation plays an important role in the pathogenesis of MetS, and numerous inflammatory cytokines have been shown to be elevated in patients with MetS [31]. TTP may act as an anti-MetS protein, as part of a homeostatic response to limit inflammation [32,33].

We found significant differences between males and females in serum TTP levels in our study cohort. Previous studies have shown sex differences in ZFP36 mRNA levels, while Vohl et al. have found that ZFP36 mRNA levels in non-diabetic, severely obese men were 3.7-fold higher in omental adipose tissues compared to abdominal subcutaneous adipose tissues [34]. In contrast, Bouchard et al. have found, in women, increased levels of ZFP36 mRNA in subcutaneous adipose tissue by twofold in omentum fats [35]. These differences could be a result of sex-specific variations of the ZFP36 gene expression upon cellular stimuli, or might be also ascribed to differences in the composition of the adipose tissues.

Our results indicated that serum TTP levels had a significant positive relationship with insulin and HOMA-IR in males with MetS, but not in women with MetS. On the contrary, Bouchard et al. reported that TTP mRNA levels were inversely correlated with HOMA-IR and fasting insulin levels in visceral adipose tissue of women. The authors suggested a protective role of TTP against IR and diabetes in omental adipose tissue [35]. Previous studies have demonstrated an upregulation effect of insulin on TTP mRNA and TTP protein in 3T3-L1 adipocytes [36,37]. Furthermore, Wang et al. demonstrated increasing effects of insulin on TTP when used in higher rather than lower glucose concentrations in 3T3-L1 cells culture media [38]. Whether TTP improves IR or not remains controversial and should be investigated in trials.

The present results showed high levels of serum TTP along with high levels of measured inflammatory cytokines, indicating that TTP could not display a downregulation of these measured inflammatory cytokines in MetS. In this regard, numerous studies have demonstrated that TTP activity is modulated through its phosphorylation, which controls its ability to bind and lead the target ARE-mRNAs for degradation [18,39,40,41]. To support this, Marchese et al. found that TTP phosphorylation through the mitogen-activated protein kinase (MAPK) p38 pathway inhibits the recruitment of the CAF1 deadenylase complex and prevents TTP from degrading ARE-mRNAs [33]. Remarkably, TTP phosphorylation through MK2 kinase did not affect the activity of TTP to bind ARE-mRNAs, proposing another effect of phosphorylation on the interaction of TTP with de-adenylase [42]. Therefore, it can be reasoned that the stimulus-dependent induction of TTP expression followed by its phosphorylation and subsequent accumulation of TTP in an inactive form can modulate the ability of TTP to promote ARE-mRNAs mediated degradation during the inflammatory response [41]. Moreover, studies have demonstrated that the TTP protein is abundantly expressed in cardiovascular disease at inflammation sites where it was expected to play its role as an anti-inflammatory protein at both the transcriptional and the posttranscriptional level [43]. TTP was also strongly expressed in the inflamed synovium of rheumatoid arthritis, which prompted the investigators to wonder why TTP failed to downregulate TNF expression and other inflammatory markers [44].

The present results showed a significant positive relationship between TTP and adiponectin in males with MetS. Adiponectin, the most abundant adipokine with substantial roles in lipid metabolism and the sensitization of insulin, along with accumulating evidence suggesting antitumor properties for adiponectin [45,46,47]. Recent studies have shown the antitumor activity of adiponectin, as globular adiponectin was found to suppresses B-cell lymphoma-2 apoptosis proteins’ expression and increased the expression of TTP in macrophages. Moreover, knocking-down TTP has repealed the effect of globular adiponectin in the suppression of B-cell lymphoma-2 apoptosis proteins’ expression [48,49]. Together, these findings propose a synergistic effect for TTP and adiponectin in the modulation of inflammation.

The authors acknowledge several limitations. The sample size in the present study was relatively small, and the members of the MetS group were significantly older than controls, making statistical adjustments necessary to compensate for differences while adding further stringency to the results. In addition, this is a cross-sectional study, and thus the potential causality between TTP levels and other parameters, particularly the inflammatory markers, cannot be assessed. Follow-up studies with better matched-controls are needed, in which TPP and inflammatory markers at multiple time points are required to ascertain if TTP levels are the cause of the increase or decrease of inflammatory markers in MetS patients. Nevertheless, the present study sheds new light, for the first time, on circulating TTP levels between individuals with or without MetS, as well as the relationship between TTP and the inflammatory state associated with MetS and its components.

## 5. Conclusions

Serum levels of TTP were higher in MetS patients than in controls, and associated with inflammatory markers, fasting insulin, HOMA-IR, and adiponectin, suggesting that TTP could play a role in the glycemic control and modulation of the associated inflammatory state in MetS. While the results at this point remain suggestive, TTP maybe a promising novel biomarker for MetS.

## Figures and Tables

**Table 1:** Clinical characteristics of the study participants.

Parameters	All	Control	MetS	*p*-Value	Adjusted for Age
N	200 (94/106)	100 (50/50)	100 (44/56)	-	-
Age (years)	38.6 ± 8.3	35.4 ± 7.9	41.9 ± 7.2	-	-
TTP (pg/mL)	242.9 (124.7–330.9)	150.9 (68.2–280.9)	288.1 (229.3–386.8)	<0.001	<0.001
Leptin (pg/mL)	145.3 (44.6–609)	111.4 (56.1–399)	159.7 (33.9–945.6)	0.59	0.78
TNF-α (pg/mL)	1.1 (0.5–1.7)	0.5 (0.2–1.1)	1.5 (0.9–1.9)	<0.001	<0.001
IL-1β (pg/mL)	0.96 (0.5–1.6)	0.5 (0.4–0.9)	1.4 (1.0–3.5)	<0.001	<0.001
CRP (ng/mL)	3242 (961–6009)	1961 (573.6–4387.9)	4452 (1727.9–6191)	<0.001	<0.001
Adiponectin (ng/mL)	10,872 (5173.9–20,176)	12,904 (4974–22,843)	10,045 (5439–16,574)	0.18	0.66
Insulin (µIU/mL)	10.9 (5.2–23.1)	8.2 (4.4–14.8)	17.2 (7.1–43.8)	<0.001	0.001
HOMA-IR	2.8 (1.2–6.8)	1.8 (0.9–3.9)	5.2 (1.8–11.3)	<0.001	-
HOMA-B	35.3 (14.8–81.2)	28.9 (14.2–53.8)	50.8 (18.3–141.1)	0.005	-

Note: Data presented in mean ± SD and median (25th–75th) percentiles. An independent *t*-test and a Mann–Whitney U test were conducted. *p*-value significant at *p* < 0.05, 0.01 level.

**Table 2:** Comparison of clinical characteristics between males and females.

Parameters	Males			Females		
N	Control	MetS	*p*-Value	Control	MetS	*p*-Value
	50	44		50	56	
Age (years)	35.2 ± 7.8	41.7 ± 7.1	<0.001	35.6 ± 8.2	42.3 ± 7.4	<0.001
TTP (pg/mL)	95.3 (43.4–187.6)	286 (221–372.6)	<0.001	231.2 (127.9–315.7)	288.9 (237.8–397.1)	0.007
Leptin (pg/mL)	149.9 (91.9–349.8)	119.6 (25.8–648.6)	0.75	93.8 (54.3–409.6)	176.8 (42.1–1706)	0.26
TNF-α (pg/mL)	0.21 (0.1–0.24)	1.2 (0.8–1.6)	<0.001	0.6 (0.4–1.2)	1.5 (0.9–2.0)	<0.001
IL-1β (pg/mL)	0.43 (0.21–0.54)	1.3 (1.1–2.7)	<0.001	0.8 (0.4–1.4)	1.6 (1.0–4.7)	<0.001
CRP (ng/mL)	1854.3 (702.5–4114)	4570 (1890–6147)	0.001	2194 (517.3–5119)	4378 (1466–6222)	0.004
Adiponectin (ng/mL)	14,892 (7313–20,878)	10,504 (5811–17,376)	0.07	9235 (2280–24,569)	9721 (4481–14,499)	0.91
Insulin (µIU/mL)	11.6 (6.5–18.9)	17.2 (5.6–52.0)	0.18	5.7 (3.6–10.9)	17.2 (7.2–35.9)	<0.001
HOMA-IR	2.8 (1.6–4.2)	6.2 (1.5–15.6)	0.037	1.3 (0.9–2.8)	4.6 (2.2–9.6)	<0.001
HOMA-B	42.2 (26.9–68.3)	56.1 (17.8–159.9)	0.37	17.6 (11.4–38.2)	50.8 (18.3–130.5)	0.003

Note: Data presented in mean ± SD and median (25th–75th) percentiles. An independent *t*-test and a Mann–Whitney U test were conducted. *p*-value significant at *p* < 0.05, 0.01 level.

**Table 3:** Relationship between MetS components and TTP among males and females.

	Overall		Male		Female	
	B ± SE	*p*-Value	B ± SE	*p*-Value	B ± SE	*p*-Value
Male	−0.13 ± 0.05	0.006				
Central Obesity	0.08 ± 0.05	0.14	0.17 ± 0.08	0.044	0.00 ± 0.07	0.96
Hypertension	0.02 ± 0.05	0.64	0.01 ± 0.08	0.89	0.06 ± 0.07	0.40
High Blood Glucose	0.10 ± 0.05	0.051	0.14 ± 0.08	0.11	0.06 ± 0.07	0.38
Low HDL-C	0.13 ± 0.05	0.011	0.14 ± 0.08	0.09	0.10 ± 0.07	0.14
High Triglycerides	0.11 ± 0.05	0.037	0.21 ± 0.09	0.016	0.04 ± 0.07	0.58
Adjusted R-Square	16.4		25.7		0.00	
Model *p*-value	<0.001		<0.001		0.450	

Note: Data presented as β ± Standard error obtained from multiple linear regression, *p*-value < 0.05 considered significant.

## Data Availability

The data presented in this study are available on request from the corresponding author.

## Supplementary Materials

The following are available online at https://www.mdpi.com/article/10.3390/biology10060550/s1, Table S1: TTP concentration according to MetS components and gender; Table S2: Correlation of TTP with other parameters.
