# Supplementary material for: Tristetraprolin, Inflammation, and Metabolic Syndrome in Arab Adults: A Case Control Study
Source: Biology (Basel). 2021 Jun 18;10(6):550. doi: 10.3390/biology10060550 (PMC8235193; doi:10.3390/biology10060550)
Supplement: Supplementary file 1 [file biology-10-00550-s001.zip › biology-1228479-supplementary.pdf]

Supplementary Table S1. Anthropometric and clinical characteristics of the study participants.

Parameters	All	Control	MetS	*p*-Value	*p*-Value Adjusted for Age
N	200 (94/106)	100 (50/50)	100 (44/56)		
BMI (kg/m^2)	29.8 ± 5.5	27.7 ± 5.2	31.8 ± 5.1	<0.001	<0.001
Waist (cm)	96.5 ± 17.6	87.6 ± 14.9	105.6 ± 15.5	<0.001	<0.001
Hips (cm)	106.7 ± 13.6	104.8 ± 12.9	108.5 ± 14.2	0.053	0.10
Waist-Hip Ratio	0.91 ± 0.16	0.84 ± 0.11	0.98 ± 0.17	<0.001	<0.001
Systolic BP (mmHg)	124.3 ± 15.7	118.0 ± 11.7	130.6 ± 16.6	<0.001	<0.001
Diastolic BP (mmHg)	74.9 ± 11.1	70.9 ± 9.2	78.9 ± 11.4	<0.001	<0.001
Glucose (mmol/L)	5.9 ± 1.9	5.3 ± 1.1	6.7 ± 2.2	<0.001	<0.001
Total Cholesterol (mmo/L)	5.3 ± 1.3	5.2 ± 1.0	5.5 ± 1.6	0.19	0.37
LDL-C (mmol/L)	3.3 ± 1.2	3.4 ± 0.9	3.3 ± 1.4	0.87	-------
HDL-C (mmol/L)	1.1 ± 0.3	1.2 ± 0.3	1.03 ± 0.3	<0.001	<0.001
Triglycerides (mmol/L)	1.8 (1.1-2.4)	1.2 (0.9-1.5)	2.3 (1.9-2.8)	<0.001	<0.001

Note: Data presented in mean ± SD and median (25th–75th) percentiles. Independent T-Test and Mann-Whitney U test was done. *P*-value significant at $p < 0.05$, 0.01 level.

Supplementary Table S2. Correlation of TTP with other parameters.

Parameters	All (N = 200)			Males (N = 94)			Females (N = 106)		
	All	Control	MetS	All	Control	MetS	All	Control	MetS
Age (years)	0.18*	0.04	−0.02	0.25*	0.38	0.06	0.09	0.02	−0.08
Height (cm)	−0.1	−0.02	−0.14	0.14	0.21	−0.23	−0.1	0.13	−0.14
Weight (kg)	0.18*	0.17	−0.13	0.33**	0.41**	−0.47**	0.075	−0.11	0.07
BMI (kg/m2)	0.27**	0.24*	−0.02	0.35**	0.42**	−0.31*	0.09	−0.21	0.15
Waists (cm)	0.21**	0.01	−0.03	0.33**	0.23	−0.17	0.19	−0.07	0.09
Hip (cm)	0.13	0.19	−0.04	0.29**	0.36*	−0.17	−0.9	−0.28	0.04
WHR	0.15*	−0.2	0.10	0.25*	−0.04**	−0.04	0.20*	0.13	0.03
SBP (mm Hg)	0.09	−0.10	−0.06	0.18	0.11	−0.27	0.05	−0.26	0.02
DBP (mm Hg)	0.19**	0.07	0.02	0.13	0.38	−0.19	0.19	0.02	0.15
Glucose (mmol/L)	0.25**	0.19	0.09	0.40**	0.1	0.23	0.18	0.26	0.003
T. Cholesterol (mmo/L)	0.06	0.13	−0.07	0.03	0.23	−0.25	0.09	0.08	0.10
LDL-C (mmol/L)	0.04	0.13	−0.04	−0.012	0.18	−0.22	0.10	0.141	0.10
HDL-C (mmol/L)	−0.15*	−0.03	−0.1	−0.50**	−0.12	−0.48**	−0.07	−0.002	0.01
Triglycerides (mmol/L)	0.21**	0.07	−0.07	0.40**	0.21	−0.01	0.10	−0.17	−0.11
Leptin (pg/ml)	0.10	0.04	0.16	0.11	0.16	0.20	0.10	0.03	0.13
TNF-α (pg/ml)	0.18*	0.003	0.11	0.30*	−0.15	−0.01	0.09	−0.16	0.20
IL-1β (pg/ml)	0.31**	0.25*	0.02	0.41**	0.16	0.15	0.17	0.16	−0.06
CRP (ng/ml)	0.18*	0.15	−0.16	0.36**	0.34*	−0.21	0.03	−0.01	−0.14
Adiponectin (ng/mL)	0.1	0.07	0.13	0.2	0.23	0.36*	0.002	0.00	0.05
Insulin (µIU/mL)	0.18*	−0.01	0.17	0.25*	0.05	0.37*	0.17	0.09	−0.15
HOMA-IR	0.24**	0.04	0.18	0.31*	0.05	0.40*	0.23	0.19	−0.13
HOMA-B	0.08	−0.5	−0.4	0.15	0.14	0.09	0.08	−0.02	−0.16

Note: Data presented as coefficient (R); * denotes significance at 0.05 level; ** denotes significance at 0.01 level.
